# Reducing infant and child mortality: assessing the social inclusiveness of child health care policies and programmes in three states of India

**DOI:** 10.1186/s12889-023-15812-7

**Published:** 2023-06-15

**Authors:** Madhumita Bango, Soumitra Ghosh

**Affiliations:** 1 Research Scholar, School of Health Systems Studies, Tata Institute of Social Sciences, V.N. Purav Marg, Deonar, Mumbai, Maharashtra-400088 India; 2 Centre for Health Policy, Planning, and Management, School of Health Systems Studies, Tata Institute of Social Sciences, Mumbai, V.N. Purav Marg, 400088 India

**Keywords:** Infant, Child, Mortality, Inequalities, Caste, NFHS

## Abstract

**Background:**

Even though the overall infant mortality rate and child mortality rate have considerably declined in India, the marginalised groups-Scheduled Caste, and Scheduled Tribe, continue to have higher mortality rates. This study looks at the changes in IMR and CMR amongst disadvantaged and advanced social groups at the national level and in three states of India.

**Data and methods:**

Data from five rounds of National Family Health Survey spanning nearly three decades have been used to measure IMR and CMR according to the social groups for India and for selected states-Bihar, West Bengal, and Tamil Nadu. The relative hazard curves were drawn to understand which social group’s children face a higher risk of dying in the first year of birth and between first year and age four in those three states. Further, a log-rank test was applied to examine whether the differences between the survival curves or distributions of the three social groups are statistically significant. Finally, the binary logit regression model was applied to investigate the effect of ethnicity, and other socio-economic and demographic covariates on the risk of infant and child deaths (1–4 years) in the country and in selected states.

**Results:**

Hazard curve shows the probability of death within one year of birth was highest among the children belonging to the ST families, followed by SCs in India. And, the CMR was found to be higher amongst the STs compared to all other social groups as well at the national level. While Bihar had a remarkably high infant and child mortality rates, Tamil Nadu possessed the lowest child death rates irrespective of class, caste, and religion. The regression model revealed that most of the caste/tribe gaps in infant and child deaths might be attributed to the place of residence, mother’s level of education, economic status, and the number of children in a family. Notably, the multivariate analysis showed that ethnicity was an independent risk factor, when controlled for socioeconomic status.

**Conclusion:**

The study detects the persistence of significant caste/tribe differentials in infant and child mortality in India. Poverty, education and health care access issues could be the possible reasons for the premature deaths of the children from deprived castes and tribes. There is a need to critically analyse the current health programmes aimed at reducing IMR and CMR to make them attuned to the needs of the marginalised communities.

**Supplementary Information:**

The online version contains supplementary material available at 10.1186/s12889-023-15812-7.

## Background

One way to assess a country’s economic performance is by looking at its mortality indicators because they not only manifest the economic disparities but also provide important insights into the nature of social inequalities [1]. The mortality rates of India have improved considerably during the last three decades—the infant and child mortality rates decreased from 86 and 119 per thousand respectively in 1992–93 to 41 and 50 per thousand respectively in 2015–16. However, the reduction in mortality was not commensurate with the high economic growth achieved since the mid-nineties [2, 3]. Besides, the progress made on the health front has not been uniform across population segments [1]. Our findings corroborate the later study, suggesting that the health inequalities, particularly the caste and tribe differences in child mortality have exacerbated in India during the post liberalisation era i.e., from 1991–92 to 2005–06.

In India, caste and tribe are markers for socio-economic status [4–7] and their association with infant and child mortality is well-established [1, 8–10]. Note, India, and especially rural India is considered to be one of the most rigidly hierarchical societies in the world. Here, different types of social stratification sort people into groups based on factors such as caste, religion or indigenous status. The caste system bestowed many privileges on those who are at the top of the caste hierarchy and imposed many social disadvantages including sanctioning of repression by the upper castes on those at the bottom. At the lowest rung of the caste ranking were the outcastes or untouchables. They are the present-day *Dalits* or Scheduled Castes (SC). Next in the hierarchy are ‘other backward classes’ (OBC), a collection of castes, which are not the victims of untouchability but they are still socially marginalised. The top-ranked castes are known as ‘upper’, ‘forward’ or ‘general’ castes. Scheduled Tribes (ST) are the Adivasis, who were subjected to severe forms of deprivation, and discrimination for centuries. According to Census 2011 [11], SC constituted about 16.6 percent of the Indian population, and a large percentage of them live in rural areas and are mainly landless agricultural labourers. ST comprised about 8.6%, while OBCs and general castes together accounted for 71 percent of India’s total population.

**Fig. 1:**
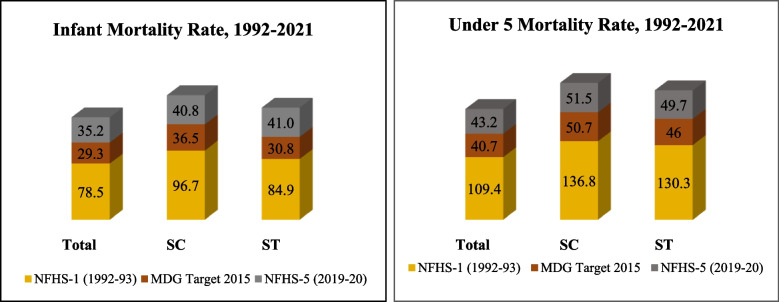
Achievements in reducing IMR and U5MR in India, 1992–2021

The recent round of data from the National Family Health Survey (NFHS)-5 (2019–21) [12] clearly shows the caste differentials in relation to child health status. The survey documents reduced access to maternal and child health services among the SCs and STs compared to the advanced castes. A decade before, the NFHS-3 (2005–06) [13] demonstrated a similar picture of poorer access and health outcomes amongst the disadvantaged social groups, which resulted in India not being able to achieve the health-related Millennium Development Goals (MDG) presented in Fig. 1.

The term ‘social inclusiveness’ in the context of policies and programmes mean designing initiatives or undertaking policy reforms in ways which would ensure that the benefits of welfare programmes reach the socially marginalised populations. It has long been recognised that socially excluded population groups face not just economic barriers but also social and cultural barriers to access health services, yet the programmes are designed and implemented disregarding the above context.

### Overview of India’s health care system reform and policy initiatives with special reference to child health care policies and programmes

India’s health sector has experienced major policy shifts since independence. Along with the overall changes in policy vision and goals, child health care policies and programmes have also evolved over time. From first five-year plan (1951–56) onward, a conscious effort was made to invest in health. In the early years of independence, the focus of health policies was mainly to develop national health programmes to control communicable diseases such as TB and Malaria, provide basic medical care, maternal and child health services (MCH) and family planning services.

Since the beginning of the plan era in 1951, population control has received disproportionate attention from the policy makers. In 1952, India became the first country in the world to launch a national programme for family planning. Health of the newborns and infants constituted a small component of this programme. After two decades, Govt. of India, in 1974, framed a national policy for children to prioritize child health, nutrition for infants and children in the pre-school age along with nutrition for nursing and expectant mothers while formulating programmes across all sectors [14]. Nevertheless, till 1977, family planning was the predominant health activity; As India’s vertical programmes like National Malaria Eradication and Family Planning Programmes achieved limited success, there were demands to critically evaluate them, which led to the incorporation of these programmes into the general district health services. Further, FP was modified into family welfare programme with MCH becoming an integral part of this programme with the understanding that reduction in infant and child mortality has a direct bearing on fertility (Govt. of India 2010). The diarrhoeal disease control programme was introduced in 1978 to prevent premature deaths of Under-5 children due to dehydration caused by diarrhea. In the same year, the Expanded Programme on Immunisation (EPI) was initiated to reduce morbidity and mortality from six vaccine preventable diseases, namely Diptheria, Pertusis, Tetanus, Polio and Childhood TB.

The vision of first National Health Policy (NHP), 1983 was to reduce IMR, NMR and CMR by the year 2000. In order to improve child survival rates, the government stressed on child health care programmes and particularly on childhood vaccination. The Government of India introduced Universal Immunisation Programme (UIP) in 1985 to remedy the shortcomings of EPI, The UIP was implemented in the country in a phased manner and was extended to the whole of India by the year 1990. Recognising the holes in the health care programmes for failing to adequately address high maternal mortality, a Child Survival and Safe Motherhood Programme (CSSMP), was launched by the Govt. of India. CSSMP subsumed UIP and it was implemented from 1992–93 to 1997–98 to improve the overall health of the infants, child and maternal morbidity and mortality. Besides improving the coverage of immunization services, CSSMP focused on interventions such as the Oral Rehydration Therapy, programme for control of Acute Respiratory Infection (ARI) in children, training of traditional birth attendants and strengthening of first referral units for providing emergency obstetric care to pregnant women. In order to address the unmet need for family welfare services in the country, especially among the poor and under-served, Govt. of India launched the Reproductive and Child Health (RCH) Programme for implementation in 1997 during the 9^th^ FYP period by integrating CSSMP with other reproductive and child health services. RCH adopted Integrated Management of Neonatal and Childhood Illnesses (INMNCI). INMNCI case management strategy was designed to provide high-quality treatment for major childhood diseases in resource-poor settings. Aside from clinical management of these common diseases of the children, INMNCI also encompasses interventions to prevent them.

However, it is argued that the pressures from various quarters, particularly the international aid agencies which held the neo-Malthusian views on population compelled Govt. of India to make greater budgetary allocations for programmes directly or indirectly linked to population control, leading to crowding out of financial and infrastructural resources for other health programmes [15, 16]. Techno-centric approaches such as RCH were thought to be the key intervention to fertility decline. Consequently, the already limited public health care services such as MCH care, nutritional services and communicable disease control programmes, which had the potential to reduce child mortality and morbidity and thus, contribute to fertility reduction, became marginalised to make way for the expansion of RCH services. The RCH approach has also been questioned on the grounds that it did not recognise the linkages of reproductive health, general health and socioeconomic conditions [16, 17].

Numerous studies pointed out the inadequacies of the existing child health care programmes in terms of their limited scope and for not being able to ensure access of the vulnerable population groups [4]. For example, during 1980s, immunization rate among the most poor and marginalised children who needed the vaccines most was found to be the lowest [18]. The non-availability and low-quality health care for a prolonged period were largely responsible for high mortality and unmeasurable adverse health outcomes among the vulnerable population groups [19]. According to an estimate, India lost nearly 1.5 million children aged under-5 years annually [20]. To reduce inequity in health care services, Govt. of India launched National Rural Health Mission (NRHM) [21] in 2005 and National Urban Health Mission in 2008. These were later integrated under the umbrella of National Health Mission to strengthen the public health care delivery system in the country. Although NHM has a wide scope, its prime focus was on enhancing utilisation of Reproductive, Maternal, Newborn, Child Health and Adolescent (RMNCH + A) services among poorer and disadvantaged castes and tribes for reducing the maternal and child mortality [22]. Currently, under NHM, several schemes are being implemented to improve the health of the children.

In spite of these ongoing efforts and progress achieved, recent evidence suggests that access to maternal and child health care continues to be determined by socioeconomic status. For example, children, more specifically children of mothers experiencing deprivations in dimensions such as education, wealth and caste or tribe remained least likely to be fully immunized (Mishra and Shyamala 2020). It is worth noting that since independence India’s health policies and five-year plans [23] have recognised ‘caste’ as the source of poor health outcomes. The Bhore committee, in 1946 [24], in its report stated the following: *“the poor state of India’s health, especially a section of people who were worse than the others in terms of health outcomes”*. The NHP 1983 envisioned to achieve ‘Health for all’ by rolling out a universal comprehensive primary health care services programme, which would effectively address the perennial issue of exclusion of the most marginalised populations from the benefits of public health services by the year 2000. In a similar vein, the 12th five-year plan document states that *“barriers to access would be recognized and overcome especially for the disadvantaged and those living far from facilities”*. However, despite the governments committing to making health care accessible to everyone, adequate efforts were not made to translate this goal into action. To put it in perspective, the public investment in health did not exceed beyond 1% of GDP even during the high growth years in 1990s. Barring a few changes at the programme level, there was hardly any major health sector initiative during the 90 s. The first decade of the twenty-first century was little better. The launch of NRHM is considered to be a watershed moment for the health sector, though the budgetary allocation has been inadequate [25].

Health inequality is a topic of longstanding interest in India. Historically, the health policies and programmes have tended not only to improve overall population health but also to reduce differences in health based on region/state, caste/tribe, socioeconomic status and other social determinants. This article seeks to produce evidence to assess the contributions of the above-mentioned policies to the realization of the health equity goal, specifically the caste/tribe differentials in infant and child mortality.

### Review of past studies

There is a growing body of literature, which sought to understand the social group disparities in health and health care in India. Studies observed that health outcomes among SCs and STs are strongly linked with their overall deprivations emanating from the social and economic relations of the caste system, which are created and reproduced in all spheres of life. Remoteness, poor diet, underdevelopment, illiteracy, and limited access to healthcare continue to have robust bearings on marginalized communities' health outcomes [8, 26–28]. A study by Ranjan et al. [29] concluded that the gap in infant mortality between tribal and non-tribal populations was substantial in the early months after birth, narrowed between the fourth and eighth months, and grew thereafter. Another study by Dommaraju and colleagues [30] demonstrated that children born to ‘lower’ caste families have a relatively higher risk of death and that women belonging to the lower castes have lower rates of antenatal and delivery care utilization than children and women from the upper caste families. In light of this evidence, the researchers of the above study argued for embedding pro-disadvantaged caste and tribe bias into the provisioning of maternal and child health services. Baru and colleagues illustrated that aside from facing socioeconomic backwardness, people from disadvantaged castes experience other adverse circumstances such as caste-based discrimination while accessing the health service system in India [1]. Studies also illustrate that disadvantaged castes and tribes still have unacceptably high child and adult mortality and persistently lower life expectancies than advanced castes [31, 32]. Further, SC and ST mothers accounted for almost 50 percent of all maternal deaths in the country, and their children were more undernourished compared to the rest of the population [33, 34].

The current study builds on the existing literature on caste and tribe differences in infant and child mortality. The point to be noted is that despite the flagship maternal and child health (MCH) programmes, the caste and tribe disparities in child health are still persisting. Existing studies sought to identify the determinants of infant and child mortality among social groups by focusing on the national level and not on the state level. Besides, there were limited attempts to examine the factors that affect the survival status of infants and children among depressed castes and tribes in India. As discussed earlier, there were some significant health policy developments in the past one and a half decade to improve maternal and child healthcare. The effects of these initiatives on improving the state of child health for the marginalised communities have remained mostly unexplored, and that make this research more relevant. Hence, this paper's first main contribution lies in the fact that it disaggregates childhood mortality into infant and under-five mortality and analyses the correlates of each using the latest round of NFHS (2019–21) data. The second main contribution of this paper is that it provides evidence on the effect of caste and tribe on infant and U5 mortality in three different states. Our study's findings can help to understand the factors behind the persistent gap in IMR and CMR between deprived castes and tribes and advanced caste groups in those three states of India.

## Materials and methods

### Data

This study is based on the publicly available data from the fifth round of National Family Health Survey (NFHS) conducted between 2019 and 2021. NFHS 5 was conducted under the governance of the Ministry of Health and Family Welfare (MOH&FW) and carried out by International Institute for Population Sciences. This survey is an Indian version of Demographic Health Survey (DHS). The sampling design of NFHS-5 has been developed considering NFHS-4 as the benchmark, and the need to provide estimates of population, health, and family welfare indicators at district, state/UT, and national levels with a reasonable level of precision. A stratified two-stage sampling design was adopted in rural and urban areas of the 707 districts (as on 31st March 2017) (NFHS, 2022). Within each rural stratum, villages were selected from the sampling frame using probability proportional to size (PPS) with explicit stratification based on the percentage of SC/ST population and female literacy. NFHS-5 covered 609,120 households with eligible women aged 15–49 and eligible men aged 15–54 from a subsample of PSUs/households in 30,456 primary sampling units (PSU), comprised of villages in rural areas and census enumeration blocks (CEBs) in urban areas. The selection of households was based on the sampling frame prepared from mapping and listing households in all PSUs identified across 707 districts (NFHS, 2022). NFHS data contains information of women 15–49 years who were asked questions about their sociodemographic characteristics and reproductive histories (births and death of their child, use of healthcare utilization etc.).

### Selection of states

We restricted our analysis to three states, namely- Bihar, West Bengal, and Tamil Nadu. Between these three states extreme health outcome variations and three kinds of health systems have been identified. Bihar is the third most populous state in India with around 40 percent of its population living below the poverty line [35]. In terms of demographics, the state has the third largest SC population in the country (16% of its population in 2011) whereas ST constituted only 1.35% of its population. The major health and demographic indicators of Bihar such as infant mortality rate, maternal mortality ratio, total fertility rate, etc. are much higher than the national average. Additionally, the state’s health index score is the lowest in the country and dropped further between 2015–16 and 2017–18, implying that Bihar is lagging behind other states in key parameters such as health outcomes, process indicators like immunization coverage and health care system inputs. On the other hand, Tamil Nadu is ranked the best among the high-performing states in India, next only to Kerala in terms of HDI. The state is internationally known for its health achievements, especially because it has made remarkable progress on the health front at low cost in recent decades. This could be possible because Tamil Nadu built a robust public health system with a centralised drug procurement model, which enabled it to make quality healthcare services accessible to the people.

According to the latest Human Development Report, though West Bengal improved its HDI score in 2017–18 over 2011–12, it still falls in the medium HDI category (Govt. of India 2020). In West Bengal, 14% of the people are below the poverty line compared to the national average of 23%. Further, according to Census 2011 population figures, socially excluded groups such as Dalits and Scheduled Tribes have a significant presence in West Bengal. The population share of the SCs is 23.5% in West Bengal, second only to Uttar Pradesh. And the STs account for 5.8% of population. The state’s health achievements are relatively better compared to other populous states. West Bengal has a large network of public health care facilities, which cater mainly to the lower and middle-income people.

It is worth noting that, in the year 2000, Jharkhand was carved out of the southern portion of Bihar. For NFHS 1 and 2 [36, 37], the districts of present Bihar state have been combined to make it universal for rest of the rounds (the districts under Jharkhand state have been dragged out). Only for the trend analysis, previous three rounds of NFHS data have been utilized.

### Description of the variables

The surviving status of infant and children aged 1–4 years was considered as the outcome variable of this study. Under-five mortality includes infant mortality and groups deaths caused by genetic-biological as well as socio-environmental factors. On the other hand, child mortality refers primarily to socio-environmental factors which is highly relevant for studying caste differences in mortality. For the predictors, we classified them into two categories—socio-economic, and demographic. These variables were found to be the important determinants of child mortality in the literature. Under demographic variables, we have considered the following variables: mother’s age (15–24, 25–34, and 35–49), birth order (1, 2–3, and 4 or more), sex of the child (male and female), ANC visit (no visit, partial or 1–3, 4 + visits), and place of delivery (home, institutional). Socio-economic variables are- place of residence (urban and rural), caste (SC, ST, and non-SC/STs- comprises Other Backward Classes (OBC) and general populations), religion (Hindu, Muslim, Christian, and others), wealth quantile (poor, middle, and rich), and mother’s level of education (no education, primary- 1 to 5 years of schooling, secondary- 6 to 12 years of schooling, and higher- more than 12 years of schooling).

### Statistical analysis

The individual-level data on the childbirth histories have been utilized to carry out mortality estimation. In the analysis, we have considered those births or deaths which were taken place in the preceding five years of the survey. We used simple bivariate techniques to examine the differences in outcome of the selected predictors between the SC, ST, and non-SC/ST caste categories. We tried to compare whether SC, ST, or non-SC/ST population groups had a higher risk of infant or child mortality. Nelson-Aalen analysis allows comparison of populations through their hazard curves. The relative hazard curves for SC, ST, and non-SC/ST populations were drawn to get a preliminary idea about which social groups are at higher risk for infants and child deaths in the selected states of India. When studying the various groups in terms of cumulative hazards functions, it is preferred to the Kaplan–Meier estimator [38]. We also plotted the hazard estimates for the three groups over the 12 months or 12–48 months analysis period. This graph's primary purpose is to show the pattern of variation in the risk of infant and child deaths between the caste groups. Second, a log-rank test was applied to examine whether there was a statistically significant difference between the survival curves or distributions of the three groups. The log-rank test statistic compared the estimates of the hazard functions of the SC, ST, and non-SC/ST populations at each observed time point. Finally, a binary logistic model was employed to examine the association between infant, child deaths and exposure variables.

## Results

### Levels, trends, and socio-economic and demographic variations in IMR and CMR in the selected Indian states

**Fig. 2:**
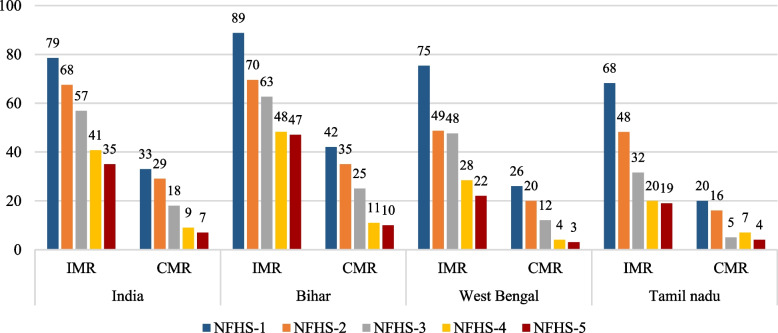
Levels and trends of IMR and CMR in Bihar, West Bengal, and Tamil Nadu, 1992–93, 1998–99, 2005–06, 2015–16, & 2019–21. Note: Authors estimated IMR and CMR for five-year period preceding the survey

Figure 2 shows the trends in IMR and CMR within the selected Indian states in India for 1992–93 to 2019–21. It is observed that though child mortality has fallen in all selected states over the study period, the pace of decline has not been uniformed everywhere. Bihar continues to top the table with highest number of infant and child deaths. However, compared to the period between 1992 and 2005, the reduction in infant and child mortality was higher in the recent years, i.e., from 2005 to 2019–21. Of the three selected states, West Bengal has relatively done well in terms of reduction of infant and child deaths. Notably, infant and child mortality rates were considerably lower in Tamil Nadu.

**Fig. 3:**
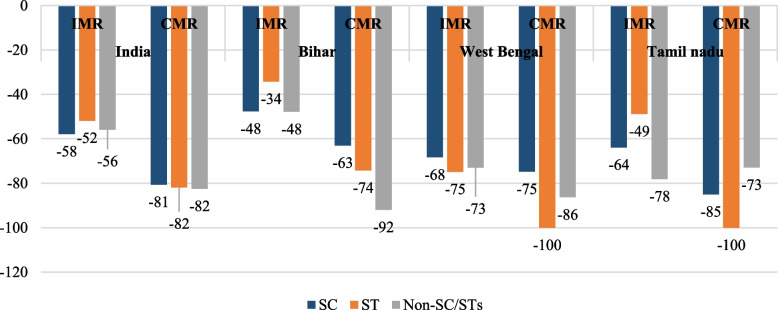
Rate of change in IMR and CMR in Bihar, West Bengal, and Tamil Nadu among social groups, 1992–93 to 2019–21 (in %). Note: Due to less number of ST population in Tamil Nadu during NFHS 1, we have taken NFHS 2 (1998–99) as a base period; not enough sample found for CMR among STs in West Bengal and Tamil Nadu in NFHS-5 (2019–21)

Figure 3 presents the rate of percentage change in infant and child mortality rates across marginalised (SC, ST) and non-marginalised groups in the selected study states from 1992–93 to 2019–21. The Fig. 3 demonstrates that the decrease in IMR and CMR was higher among the advanced castes compared to the SCs and STs during the study period for whole India, but the rate of reduction in mortality rates was different among the states. Unfortunately, in Bihar, reduction in infant deaths decreased by 48 percent among SCs which is least compared to Tamil Nadu and West Bengal. On the other hand, in West Bengal, decline in child deaths happened proportionately among all the caste categories, though the reduction was lesser among the SC population compared to the non-SC/STs and STs. However, Tamil Nadu poses the least child deaths in all the caste categories in the last two decades.

The coefficients of mortality for the target population have been shown in Table 1. The IMR and CMR were both high in rural areas compared to urban areas. The rural–urban disparity of IMR and CMR can be seen across the selected states also. In case of IMR, Bihar accounted for the highest share of infant deaths in both rural and urban areas, followed by West Bengal and Tamil Nadu. The highest IMR was observed in Bihar (48), followed by CMR (13) in rural Bihar and the least in the state of Tamil Nadu, IMR (22) and West Bengal, CMR (4). Within the different religious groups, the IMR and CMR was high among Hindus (36 and 7) respectively, followed by Muslims (34, 6) in India. The state-wise variation shows, Hindus of Bihar has a higher rate of IMR and CMR as well. Interestingly, in Tamil Nadu IMR is low among the Christian population (18), although CMR was high among the Hindus (5). The health inequity can be found among the unprivileged social classes, IMR was highest among the SC population (41) while CMR was high among the ST population (7) in India. A sporadic pattern of IMR and CMR can be seen among the SC/ST populations across different States. IMR among the SC population was highest in Bihar (47) and Tamil Nadu (25). In contrast, IMR (25) and CMR (7) was highest in West Bengal, respectively. While segregating the India population in terms of different wealth quintiles, the IMR and CMR was found to be highest among the poor sections followed by middle and rich people. Furthermore, the bio-medical covariates have shown a higher coefficient value of IMR and CMR among the mothers of lower (15–24) and higher (35–49) age-groups in India. In Bihar, IMR (54) was highest among West Bengal and Tamil Nadu. A similar picture can be seen for the educational attainment of the mothers. The IMR and CMR was high with lower education/no education level of mothers. The national figure shows the highest IMR (49) and CMR (16) among no- educated mothers. Interestingly, in West Bengal, higher rate of IMR (27) and CMR (1) was found among the mothers with primary education only. Finally, the determining factor of birth order shows, a mother with 4 or more birth order has a higher coefficient value of IMR (50) and CMR (18) across India. While Bihar poses the highest IMR (57) with lower birth order. Mothers with 4 + ANC visits show a lower rate of child deaths in India and across the states as well, while institutional delivery also seems to be an important factor for IMR and CMR in India.

**Table 1:** Infant Mortality Rates and Child Mortality Rates by mother’s socio-economic and demographic characteristics in Bihar, West Bengal, and Tamil Nadu, 2019–21

Background characteristics	India		Bihar		West Bengal		Tamil Nadu	
	**IMR**	**CMR**	**IMR**	**CMR**	**IMR**	**CMR**	**IMR**	**CMR**
**Place of residence**								
Urban	26.6	5.2	44.2	12.6	20.4	3.0	13.7	3.2
Rural	38.4	7.6	47.7	12.9	22.2	3.8	21.6	5.7
**Religion**								
Hindu	35.9	7.2	46.6	13.8	19.2	2.8	18.3	4.7
Muslim	33.6	6.1	50.3	8.4	25.6	5.2	14.4	0##
Christian	27.3	3.9	64.8#	0##	54.4	0##	17.7	0##
Other	28.7	8.1	59.9#	0##	14.1	0##	0##	0##
**Caste**								
Scheduled Caste	40.8	8.6	47.3	11.1	24.8	6.9	24.8	4.9
Scheduled Tribe	40.9	9.0	57.6	17.9	24.3	0##	31.3	0##
Non-SC/STs	32.9	5.2	46.6	7.9	19.9	3.4	14.9	4.2
**Wealth Quantile**								
Poor	44.7	11.95	48.6	15.2	23.1	5.2	32.9	16.8
Middle	33.4	6.99	51.4	5.5	20.3	1.2	19.3	4.8
Rich	23.4	4.12	32.5	5.8	17.7	0##	13.3	0.2
**Mother's age**								
15–24	40.3	5.92	54.2	8.2	24.8	3.9	17.7	3.3
25–34	32.3	7.7	40.9	10.8	17.6	3.5	18.6	5.1
35–49	36.1	15.16	54.2#	33.0#	31.2	0##	14.5	0##
**Mother's education**								
No education	49.2	16.23	54.5	17.4	23.0	6.1	37.4#	0##
Primary	43.1	8.19	46.4	15.3	27.4	1.1	25.9	0##
Secondary	32.1	6.05	43.9	7.6	22.1	1.3	20.7	6.8
Higher	19.8	3.18	21.6	5.5	7.3	0##	12.8	0.7
**Birth order**								
1	35.5	5.13	57.4	7.6	20.3	2.7	15.3	1.4
2–3	31.3	8.26	39.9	12.8	22.2	1.1	19.2	6.5
4 or more	50.4	18.43	49.3	19.8	30.8	28.7#	76.0	28.1#
**Sex of the child**								
Male	37.6	6.5	49.8	9.4	24.8	6.3	20.6	1.9
Female	32.7	7.4	44.4	16.8	18.5	0.9	15.2	7.3
**ANC visits**								
No ANC	40.4	15.02	40.7	21.7	44.6#	0##	18.3#	0##
Partial (1–3)	28.9	8.34	31.2	12.9	20.5	0##	25.0	0##
4 + ANC	19.1	4.86	29.0	6.2	13.4	2.5	10.3	6.04#
**Place of delivery**								
Home	51.2	17.38	48.6	18.3	22.6	12.9	17.4#	0##
Institutional	33.2	6.95	46.8	10.9	21.6	2.7	17.5	4.3

**Note:** #not enough sample, ##no sample found

### Cumulative hazard rates

**Fig. 4:**
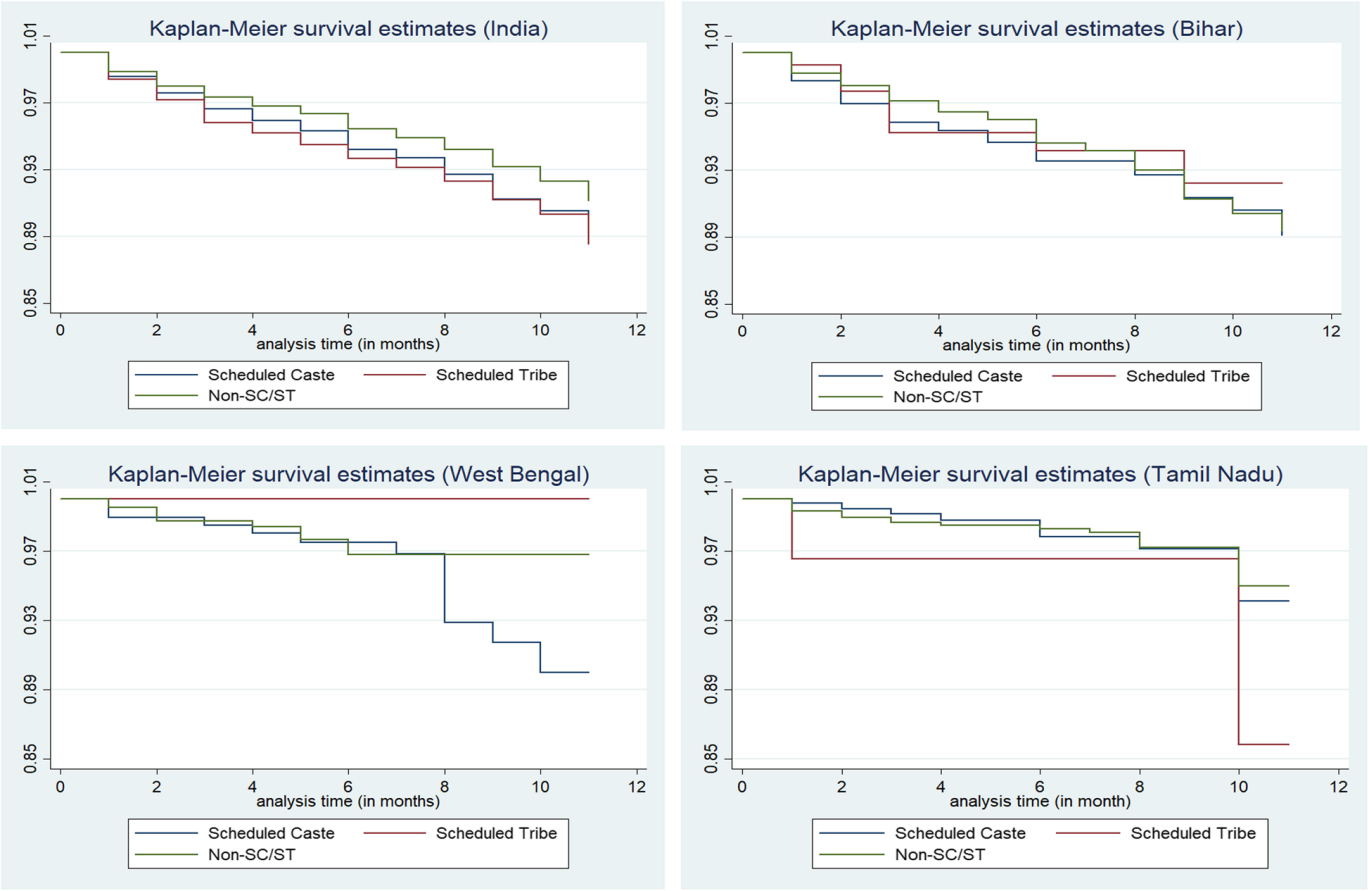
Cumulative hazard functions (infant mortality) for SC, ST, and Non-SC/ST populations in India, Bihar, West Bengal, and Tamil Nadu, NFHS-5, 2019–21. Note: not enough ST samples found in West Bengal and Tamil Nadu

Figure 4 represents the Kaplan–Meier cumulative [39] hazard curve for SC, ST, and non-SC/ST populations for India and each selected state. The curves reveal that infants of SCs and STs were at higher risk of death in their first year of lives compared to their counterparts from non-SC/ST families across India and within each selected state. Further, in Bihar, the cumulative hazards rate of both groups (SC and non-SC/ST) were found to be similar, meaning that the risk of infant death is kind of similar at the later stage while completing 12 months. In the remaining two states, the hazard rates were higher among SCs and remained higher during the first year of life. This indicates that the disparity in infant death's risk existed during the first year of life among the disadvantaged castes in the selected states. Due to the smaller size of tribal population in Tamil Nadu and West Bengal, the curve for STs is showing straight.

**Fig. 5:**
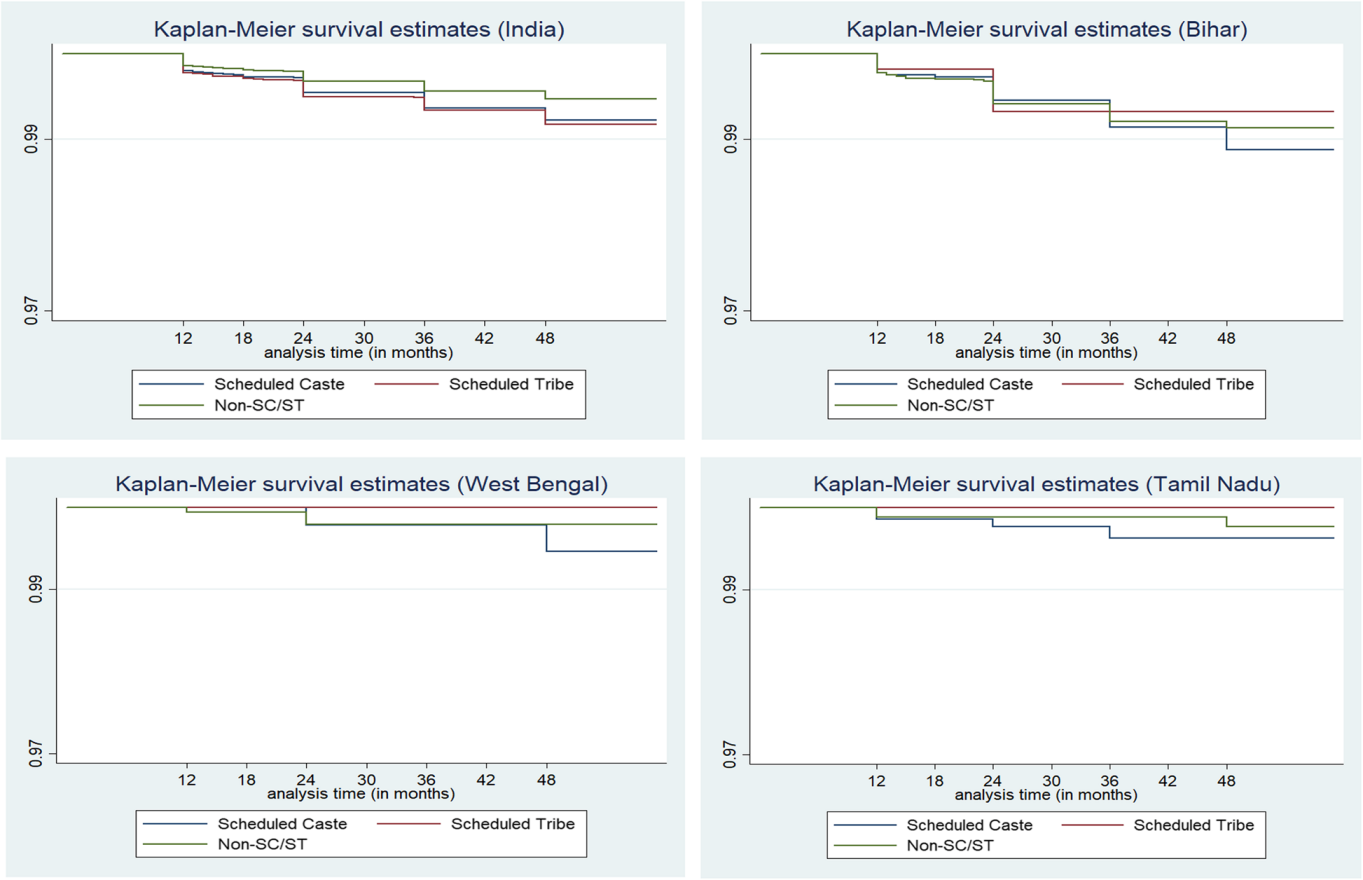
Cumulative hazard functions (child mortality) for SC, ST, and Non-SC/ST populations in India, Bihar, West Bengal, and Tamil Nadu, NFHS-5, 2019–21. Note: not enough ST samples found in West Bengal and Tamil Nadu

The cumulative hazard rate curve of child mortality (Fig. 5) depicts similar trends as infant mortality. The risk of dying before or up to four years was more among ST children, followed by the Scheduled Caste children. The curves highlight, in West Bengal, the pattern of survival chances was quite similar for the two social groups (SC and non-SC/ST). In Tamil Nadu and Bihar, the hazard rates were higher for SCs and remained that way during the first three years of life.

The log-rank test (Table 2) confirmed that children of SCs and STs in the states were at higher risk of death than non-disadvantaged groups in their first year or four years of lives as there was a statistically significant difference between the survival distributions of disadvantageous population groups and rest of the population.

**Table 2:** Log-rank test for equality of survivor functions for SC, ST and Non-SC/STs populations in India, 2019–21

	**Infant Mortality Rates**		**Child Mortality Rates**	
**Caste**	**Event Observed**	**Event Expected**	**Event Observed**	**Event Expected**
Scheduled Caste	524	466.6	192	160.8
Scheduled Tribe	562	463.7	206	161.1
Non-SC/STs	1098	1253.7	358	434.1
Total	2184	2184	756	756
**Note:**	χ2 (1) = 47.33		χ2 (1) = 31.94	
	*p* < *0.0001*		*p* < *0.0000*	

## Results from logit regression models

Table 3, 4, 5, and 6 present the odds ratios for the predictors of infant and child deaths. Model- 1 is a basic model which includes only the caste and tribe as a covariate. In the second model, in addition to caste and tribe, a set of socio-economic factors such as place of residence, religion, wealth index, and mother’s education are included. In model 3, demographic factors which are considered to be proximate determinants (such as- mother’s age, birth order, sex of the child, ANC visits, and place of delivery) are included along with caste and tribe. And model 4 is the full model, where the basic, socio-economic, and proximate factors are included. The reason behind the inclusion of these variables is to determine if the adjustments would modify the size of the effects of caste on infant or child mortality. In another words, by controlling the effects of other covariates, we can disentangle the net effect of caste/tribe on mortality in each of the selected states.

**Table 3:** Logit estimates (*p*-values) of infant and child death risk by different characteristics in India, 2019–21

Variables	Infant Mortality				Child Mortality			
	**Unadjusted OR (Model 1—basic)**	**Adjusted OR (Model 2—socio-economic)**	**Adjusted OR (Model 3—proximate)**	**Adjusted OR (Model 4—full model)**	**Unadjusted OR (Model 1—basic)**	**Adjusted OR (Model 2—socio-economic)**	**Adjusted OR (Model 3—proximate)**	**Adjusted OR (Model 4—full model)**
**Place of residence**								
Urban®								
Rural		1.12*** (0.00)		1.05** (0.03)		1.83*** (0.00)		1.75 (0.07)
**Caste**								
Non-SC/ST®								
SC	1.26*** (0.00)	1.16*** (0.00)	1.16*** (0.00)	1.11*** (0.00)	1.43*** (0.00)	1.19*** (0.00)	1.38** (0.01)	1.19 (0.20)
ST	1.05* (0.09)	1.09* (0.09)	1.05* (0.09)	0.92** (0.05)	1.56*** (0.00)	1.16** (0.05)	1.26** (0.05)	1.22* (0.10)
**Religion**								
Hindu®								
Muslim		0.95 (0.21)		0.91* (0.07)		0.87 (0.27)		0.73** (0.01)
Christian		0.67*** (0.00)		0.64*** (0.00)		1.05*** (0.00)		0.69*** (0.00)
Others		0.80*** (0.00)		0.82* (0.03)		0.92*** (0.00)		0.88** (0.03)
**Wealth index**								
Poor®								
Middle		0.72*** (0.00)		0.79*** (0.00)		0.71*** (0.00)		0.74*** (0.00)
Rich		0.57*** (0.00)		0.67*** (0.00)		0.55*** (0.00)		0.71*** (0.00)
**Mother's age**								
15–24®								
25–34			0.74*** (0.00)	0.78*** (0.00)			1.59*** (0.00)	1.73*** (0.00)
35–49			0.85*** (0.00)	0.94 (0.29)			3.00*** (0.00)	3.19*** (0.00)
**Mother's level of education**								
No education®								
Primary		0.93 (0.15)		0.98 (0.77)		0.62*** (0.00)		0.94 (0.22)
Secondary		0.69*** (0.00)		0.78*** (0.00)		0.44*** (0.00)		0.75*** (0.00)
Higher		0.43*** (0.00)		0.54*** (0.00)		0.28*** (0.00)		0.54*** (0.00)
**Birth order**								
1®								
2'-3			0.86*** (0.00)	0.84*** (0.00)			1.41** (0.02)	1.18 (0.30)
4 or more			1.37*** (0.00)	1.32*** (0.00)			1.92*** (0.00)	1.22 (0.30)
**Sex of the child**								
Male®								
Female			0.89*** (0.00)	0.89*** (0.00)			1.07 (0.50)	1.08 (0.70)
**ANC visits**								
No ANC®								
Partial (1–3)			0.77*** (0.00)	0.76*** (0.00)			0.84 (0.32)	0.85 (0.30)
4 + ANC			0.61*** (0.00)	0.61*** (0.00)			0.61*** (0.00)	0.69*** (0.00)
**Place of delivery**								
Home®								
Institutional			0.92* (0.10)	0.92** (0.01)			0.58*** (0.00)	0.88** (0.04)

**Note:** *p < 0.10, **p < 0.05, ***p < 0.005

**Table 4:** Logit estimates (*p*-values) of infant and child death risk by different characteristics in Bihar, 2019–21

Variables	Infant Mortality				Child Mortality			
	**Unadjusted OR (Model 1—basic)**	**Adjusted OR (Model 2—socio-economic)**	**Adjusted OR (Model 3—proximate)**	**Adjusted OR (Model 4—full model)**	**Unadjusted OR (Model 1—basic)**	**Adjusted OR (Model 2—socio-economic)**	**Adjusted OR (Model 3—proximate)**	**Adjusted OR (Model 4—full model)**
**Place of residence**								
Urban®								
Rural		1.29* (0.10)		1.22** (0.03)		1.78** (0.05)		1.53** (0.02)
**Caste**								
Non-SC/ST®								
SC	1.11** (0.01)	1.08** (0.03)	1.07** (0.05)	1.1** (0.05)	1.11** (0.01)	1.87** (0.05)	1.10** (0.05)	1.85** (0.03)
ST	1.32* (0.10)	1.27* (0.10)	0.82 (0.49)	0.8 (0.44)	0.86* (0.10)	0.72 (0.59)	0.56 (0.34)	0.53 (0.53)
**Religion**								
Hindu®								
Muslim		1.01 (0.95)		0.98 (0.80)		0.72 (0.29)		0.65 (0.34)
Christian		0.99 (1.0)		#		#		#
Others		1.73 (0.46)		#		#		#
**Wealth index**								
Poor®								
Middle		0.92 (0.42)		0.97 (0.63)		0.40** (0.05)		0.51* (0.07)
Rich		0.70** (0.01)		0.78 (0.28)		0.67** (0.04)		0.60 (0.47)
**Mother's age**								
15–24®								
25–34			0.67*** (0.00)	0.68*** (0.00)			2.17* (0.08)	2.32* (0.08)
35–49			1.09 (0.63)	1.12* (0.10)			5.87*** (0.00)	6.21*** (0.00)
**Mother's level of education**								
No education®								
Primary		0.85** (0.03)		0.86** (0.03)		0.81 (0.48)		0.99 (0.91)
Secondary		0.78** (0.04)		0.79* (0.07)		0.58** (0.02)		0.94* (0.08)
Higher		0.47*** (0.00)		0.53** (0.02)		0.13** (0.05)		0.48** (0.05)
**Birth order**								
1®								
2'-3			0.66*** (0.00)	0.66*** (0.00)			0.91* (0.08)	0.82* (0.07)
4 or more			0.99* (0.09)	0.98* (0.09)			1.21 (0.73)	1.01 (0.99)
**Sex of the child**								
Male®								
Female			0.88 (0.20)	0.88 (0.21)			1.40 (0.25)	1.40 (0.26)
**ANC visits**								
No ANC®								
Partial (1–3)			0.76** (0.03)	0.76** (0.03)			0.91* (0.06)	1.21 (0.62)
4 + ANC			0.75** (0.05)	0.75* (0.06)			0.47* (0.09)	0.52** (0.04)
**Place of delivery**								
Home®								
Institutional			0.91* (0.10)	0.92* (0.10)			0.79 (0.45)	0.83 (0.57)

**Note:** *p < 0.10, **p < 0.05, ***p < 0.005; #—not enough sample

**Table 5:** Logit estimates (*p*-values) of infant and child death risk by different characteristics in West Bengal, 2019–21

Variables	Infant Mortality				Child Mortality			
	**Unadjusted OR (Model 1—basic)**	**Adjusted OR (Model 2—socio-economic)**	**Adjusted OR (Model 3—proximate)**	**Adjusted OR (Model 4—full model)**	**Unadjusted OR (Model 1—basic)**	**Adjusted OR (Model 2—socio-economic)**	**Adjusted OR (Model 3—proximate)**	**Adjusted OR (Model 4—full model)**
**Place of residence**								
Urban®								
Rural		1.73** (0.02)		1.09* (0.08)		1.14** (0.02)		#
**Caste**								
Non-SC/ST®								
SC	1.28** (0.03)	1.60* (0.09)	1.00** (0.01)	1.13* (0.07)	1.74* (0.06)	7.08 (0.21)	#	#
ST	1.43** (0.24)	1.56 (0.26)	1.60 (1.24)	1.68 (0.20)	#	#	#	#
**Religion**								
Hindu®								
Muslim		1.61* (0.10)		1.27* (0.05)		1.76* (0.05)		#
Christian		4.16** (0.03)		3.75* (0.09)		#		#
Others		1.62 (0.64)		#		#		
**Wealth index**								
Poor®								
Middle		0.73 (0.30)		0.99 (0.98)		0.73 (0.29)		#
Rich		0.62** (0.02)		0.96 (0.94)		#		#
**Mother's age**								
15–24®								
25–34			1.09 (0.79)	1.08 (0.81)			#	#
35–49			2.18* (0.10)	2.18* (0.10)			#	#
**Mother's level of education**								
No education®								
Primary		1.30* (0.05)		1.35 (0.48)		0.69 (0.72)		#
Secondary		0.73** (0.04)		0.76 (0.51)		0.19 (0.11)		#
Higher		0.60** (0.04)		0.66 (0.56)		#		#
**Birth order**								
1®							#	
2'-3			1.22* (0.05)	1.22 (0.53)			#	#
4 or more			1.25 (0.70)	1.26 (0.69)			#	#
**Sex of the child**								
Male®								
Female			0.74 (0.25)	0.73 (0.24)			#	#
**ANC visits**								
No ANC®								
Partial (1–3)			0.63** (0.03)	0.59** (0.03)			#	#
4 + ANC			0.53* (0.10)	0.51* 90.10)			#	#
**Place of delivery**								
Home®								
Institutional			0.96 (0.90)	0.94 (0.93)			#	#

**Table 6:** Logit estimates (*p*-values) of infant and child death risk by different characteristics in Tamil Nadu, 2019–21

Variables	Infant Mortality				Child Mortality			
	**Unadjusted OR (Model 1—basic)**	**Adjusted OR (Model 2—socio-economic)**	**Adjusted OR (Model 3—proximate)**	**Adjusted OR (Model 4—full model)**	**Unadjusted OR (Model 1—basic)**	**Adjusted OR (Model 2—socio-economic)**	**Adjusted OR (Model 3—proximate)**	**Adjusted OR (Model 4—full model)**
**Place of residence**								
Urban®								
Rural		1.59** (0.04)		1.27** (0.04)		1.32** (0.04)		1.51* (0.07)
**Caste**								
Non-SC/ST®								
SC/ST	1.83*** (0.00)	1.59** (0.02)	1.89** (0.02)	1.93** (0.02)	1.79** (0.03)	1.23** (0.03)	3.55 (0.39)	2.37** (0.03)
**Religion**								
Hindu®								
Muslim		1.25 (0.67)		1.78 (0.36)		#		#
Christian		1.15 (0.77)		0.87 (0.85)		#		#
**Wealth index**								
Poor®								
Middle		0.73** (0.02)		0.86* (0.07)		0.78 (0.75)		0.55 (0.56)
Rich		0.67** (0.03)		1.06* (0.09)		0.31 (0.25)		0.20 (0.24)
**Mother's age**								
15–24®								
25–34			0.81 (0.69)	0.88 (0.71)			0.65 (0.65)	0.73 (0.64)
35–49			0.82* (0.07)	0.91* (0.07)			#	#
**Mother's level of education**								
No education®								
Primary		0.83 (0.78)		0.59 (0.82)		#		#
Secondary		0.69 (0.49)		0.3 (0.66)		0.70 (0.53)		0.60 (0.61)
Higher		0.52 (0.24)		0.15 (0.89)		0.56 (0.30)		#
**Birth order**								
1®								
2'-3			1.47** (0.02)	1.36** (0.03)			1.21* (0.08)	1.20 (0.85)
4 or more			3.69** (0.05)	3.21* (0.09)			#	#
**Sex of the child**								
Male®								
Female			0.83 (0.50)	0.83 (0.50)			0.73 (0.55)	1.69 (0.57)
**ANC visits**								
No ANC®								
Partial (1–3)			0.66** (0.05)	0.64** (0.05)			#	#
4 + ANC			0.42* (0.09)	0.4* (0.09)			#	#
**Place of delivery**								
Home®								
Institutional			0.25*** (0.00)	0.24*** (0.00)			#	#

**Note:** *p < 0.10, **p < 0.05, ***p < 0.005; since there is not enough sample found for STs, SCs and STs have been combined

The significant effect of caste/tribe can be seen on infant and child deaths at national and state level in the four models presented in the Tables 3–6. As per the results of the first model, SC (OR:1.26, *p* < 0.005; OR:1.27, *p* < 0.005) and ST (OR:1.05, *p* < 0.10; OR:1.19, *p* < 0.005) children had a higher likelihood of dying compared to non-SC/ST children at the national level (Table 3). However, other models (2, 3 and 4) show relatively smaller influence of caste and tribe on infant and child mortality. As depicted in Tables 4–6, across states, infants born to disadvantaged social groups-ST and SC were more likely to die relative to those born to non-SC/ST communities. For instance, results from the basic models (caste adjusted OR) suggest that tribal babies were over 1.3 and 1.4 times more likely to die before their first birthday than those born in non-SC/ST families in Bihar and West Bengal respectively. However, in case of Bihar and West Bengal, the addition of socioeconomic, demographic and health service utilisation factors in models 4 and 5 attenuated the mortality risk for the infants of scheduled caste mothers by a certain extent. Furthermore, controlling for the above-mentioned variables erased differences in mortality risk between infants of ST and non-SC/ST families. The results regarding caste and tribe differences in infant mortality are more striking for Tamil Nadu. According to the basic model, babies of SC and ST families were 1.8 times more likely to die before completing age one than those from non-SC/ST families. Interestingly, the remaining models show that even after controlling for other factors, effect of caste and tribe did not wane. On the contrary, the values of OR increased.

The findings are broadly similar when we explored the effects of caste and other covariates on child mortality. These results imply that caste disparities in infant and child mortality could be largely explained by the existing structural and health service inequalities in these two eastern states of India. But Tamil Nadu is clearly at odds with Bihar and West Bengal. In socioeconomic, demographic and health service utilisation adjusted analyses, relative to non-SC/STs, ST and SC children were twice as likely to die as children from non-SC/ST families.

In other words, the above results suggest that differences in socio-economic status and proximate indicators are strongly associated with disparities between advanced and deprived castes and tribes in infant in Bihar and West Bengal, whereas Tamil Nadu shows a different picture with no such significant changes in mortality risk after introducing socio-economic and health service determinants. This tends to support the hypothesis that the caste and tribe have an independent effect on infant and child mortality.

All models showed that economic status was a significant risk factor for infant and child mortality at the national level. Children from the rich households (OR: 0.57, *p* < 0.005; OR: 0.55, *p* < 0.005) were less likely to die than their counterparts from the poor households. We also find that place of delivery, mother’s age, birth order, education and ANC use were important predictors of infant and child mortality at the national level. However, full models show that some of the above covariates such as place of delivery and wealth index were not found to have statistically significant association with child mortality for all three selected states. For example, full models show that wealth index was not associated with child mortality for all three states including Tamil Nadu. Another important finding is that higher utilization of ANC services was found to be significantly and consistently associated with higher survival chances for infants and children across states.

The results also revealed that children were less likely to die when their mothers had completed secondary (OR: 0.69, *p* < 0.005; OR: 0.66, *p* < 0.005) or higher (OR: 0.54, *p* < 0.005; OR: 0.54, *p* < 0.005) level of education compared with those children born to mothers with no formal education. Note, mother’s education did not show any statistically significant effects on infant or child mortality in West Bengal and Tamil Nadu. The likelihood of lower infant mortality increased when mothers were opting for institutional delivery over home delivery in India. In case of Tamil Nadu, model 4 shows that babies born in institutions were significantly less likely to die than those born in homes (OR = 0.24).

## Discussion

This article examined the trends and socioeconomic differentials in infant and child mortality rates by social groups, especially SC and ST for selected states of India during the period between 1992–93 and 2019–21, when the country’s child health care policies and programme evolved over the time and major initiatives such as NHM were undertaken. To explore how survival outcomes vary for babies born to mothers with different caste/tribe backgrounds, survival estimates were estimated for infants and children aged 1–4 years who were born to disadvantaged castes or tribes (SC or ST) versus those born to non-SC/ST mothers.

Our study highlights substantial inequalities in infant and child mortality between castes and tribes (SC, ST, and non-SC/STs) during the study period. What is disconcerting to note is that the caste and tribe differences in IMR and CMR exacerbated in the last one and a half decades. Among the selected states, Bihar experienced the highest mortality burden in terms of infant and child mortality rates, and though the mortality rates diminished over time, the rate of decline was found to be lowest for the Dalits (SCs) (Fig. 3). The findings from regression analysis at the national level too revealed that children from the deprived castes and tribe backgrounds had higher likelihood of deaths than those from non-deprived castes and tribes. It is worth noting that even after controlling for the children’s socioeconomic status and their mothers’ demographic characteristics, SC and ST children’s risk of death were still statistically different (higher) than non-SC/ST children. Similarly, among the infants, the differences in risk of mortality persisted after correction for socioeconomic status. In another words, the multivariate analysis shows that ethnicity was an independent risk factor, when controlled for socioeconomic status.

In fact, the effect of ethnicity on IMR and CMR was observed across all selected states, and interestingly, Tamil Nadu, which is one of India’s most developed states and widely celebrated for its welfare schemes for advancing social justice has put Bihar and West Bengal behind in this matter. The odds ratios depict that SCs and STs are at a marked disadvantage as regards to infant and child mortality. That the Tamil children from SCs and STs face considerably higher mortality risk than their fortunate counterparts from non-SC/ST families are also supported by recent literature. Mishra, Rinju and Panda [40], while ranking the states using an index of relative disadvantage for different social groups with respect to the prevalence of mortality at household level noted that Tamil Nadu features at the top of the table, just below Punjab with the greatest disadvantage for SCs. According to the authors, the reasons are not very clear and can only be ascribed to poverty, education and health care access issues.

Other important findings of this study, which are in consonance with the above results, emerged from the survival analysis. The hazard curves provided evidence that the risk of deaths was indeed far greater for babies born to mothers from SC and STs and their plight was uniform across selected states. However, for those who completed 12 months, West Bengal clearly stands out in the sense that the pattern of mortality risks was found to be very similar for all regardless of whether they are from SC/ST or non-SC/ST families. But in the case of Tamil Nadu and Bihar, the risks of non-survival were much higher for SC children and that stayed unchanged during the first five years of life.

Expectedly, in Tamil Nadu, where epidemiologic transition is most advanced, mortality risk was lowest among non-SC/STs with relatively higher risk among SC children. Unfortunately, due to small sample size, not much insights could be gained into the mortality risks of tribal children in Tamil Nadu and West Bengal. Ranjan, Dwivedi and Brajesh [41] studied infant mortality in tribal population in India. that the difference in infant mortality between tribal and non-tribal populations was substantially higher in the early months after birth among tribals; it narrowed between the fourth and eighth months; this is maybe due to inaccessibility or unavailability of postnatal care services and low levels of socio-economic development [42].

The multivariate analysis of the present study revealed a statistically significant association between mother’s level of education, household wealth, and infant and child deaths. As predicted, the risk of infant and child deaths decreases with the increase of mother’s educational attainment. Bora, Raushan and Lutz [8] explained that educated mothers are more likely to develop good health-seeking behavior, like- utilization of healthcare services, feeding and care practices, etc., which in turn help produce better health outcomes for children and mothers both. A similar trend has been observed for household wealth. Our study found that the infant mortality rate for the poorest quintile was three times higher than that for the wealthiest income quintile. Also, numerous previous studies established alike conclusions [43–46].

Mother’s characteristics like- birth order, sex of the child also have a strong association with child deaths. Mothers with a greater number of children are more likely to experience infant and child deaths irrespective of the caste category. On the contrary, few earlier studies highlighted that after controlling for factors such as mother’s age and child’s birth weight, the child’s birth order does not affect its mortality for the first six months of life [47]. This could be because women’s behavior towards postnatal child care and utilization of modern healthcare facilities do not change for higher-order births [48, 49]. Moreover, the risks of mortality before the age of four years are higher in girls than in boys. However, for tribes, the rural–urban difference in the risk of infant death was not significant. This may be because more than 95 percent of tribal populations reside in rural areas, and they have homogeneously low access to and utilization of maternal and antenatal/postnatal care services [28, 42, 50–52].

Despite continuing efforts by the government to recompense the caste system's effects through a reservation system, caste remains a significant line of social division in India. However, people from deprived castes continue to face multiple disadvantages compared to the rest of the population [53, 54] and still have lower socioeconomic development indicators than the rest of the population [55]. It may also be noted that initiatives like National Health Mission (NHM) made an attempt to consolidate the public health care infrastructure in the rural areas. Schemes such as JSY and JSSK (Janani Sishu Suraksha Karyakaram) under NHM are being implemented to improve the IMR, U5MR, and MMR by conditional cash transfer for institutional deliveries to the women from caste groups and poor households. Though these efforts bore fruits and institutional delivery rates and mortality indicators improved among the SCs and STs, our study confirms the persistence of caste disparities in IMR and CMR even in the NHM period.

This study has quite a few strengths. This is the study that describes mortality differentials between the deprived and advanced caste/ethnic groups and along with the caste and class differentials, we attempted to explore the extreme regional disparities as well, using the recently released NFHS data (2019–21). Further, multivariate analysis recognized the key drivers of infant and child deaths in India.

## Conclusions

To conclude, there is substantial caste and tribe differentials in infant and child mortality in India. And the effect of ethnicity on infant and child mortality varies across states. However, rural–urban differences in mortality rates have also been recognized. Factors like- place of residence, household wealth, mother’s education, birth order, and sex of the child are significantly associated with child mortality in India. The study findings imply policy actions to prioritize the needs of the marginalized population groups in the provision of maternal and child health services in India. One of the most significant key areas of the policies should be equitable access of public services including health care; this would mean affirmative action to reach the poorest and minimizing disparity on account of gender, poverty, caste, disability, and other forms of social exclusion, and geographical barriers. However, although the issue of the differentials in India's mortality patterns by class and caste, received more attention over the last few decades in health policies and programmes, the actual public spending on health has not seen as much increase as it warranted to redress the social inequalities in health. And therefore, these programmes had limited success. Hence, it is essential to review these policies and design them in ways that they become attuned to the needs of the marginalized communities. This would improveMCH indicators improve in an equitable manner, and help achieve health-related SDG goals. Additionally, the federal government and states need to find ways to ensure that pregnant mothers and children, particularly those from the marginalized sections have access to adequate quality food, clean water and toilets. Additionally, greater efforts must be made to educate the marginalized communities regarding the importance of maternal and child health services including Ante Natal Check-up (ANC), Post Natal Check-up (PNC), and vaccination.

## Supplementary Information

**Additional file 1.**

## Data Availability

The datasets generated and/or analysed during the current study are available on the DHS website, https://dhsprogram.com/data/available-datasets.cfm

## Declarations

### Patient and public involvement

Patient and public were not involved.
