# Supplementary material for: Reducing infant and child mortality: assessing the social inclusiveness of child health care policies and programmes in three states of India
Source: BMC Public Health. 2023 Jun 15;23:1149. doi: 10.1186/s12889-023-15812-7 (PMC10268457; doi:10.1186/s12889-023-15812-7)
Supplement: Supplementary file 1 — Additional file 1. [file 12889_2023_15812_MOESM1_ESM.docx]

**Appendix**

**Table A1.** Number of observations of IMR and CMR by caste groups in Bihar, West Bengal, and Tamil Nadu, 2019-21 (NFHS-5)

| **States** | **IMR** | | | | **CMR** | | | |
| --- | --- | --- | --- | --- | --- | --- | --- | --- |
|  | **SC** | **ST** | **Non-SC/STs** | **Total** | **SC** | **ST** | **Non-SC/STs** | **Total** |
| India | 1,933 | 1,603 | 4,123 | 7,659 | 192 | 206 | 358 | 756 |
| Bihar | 293 | 43 | 634 | 970 | 31 | 3 | 67 | 101 |
| West Bengal | 40 | 12 | 46 | 98 | 13 | 0 | 3 | 16 |
| Tamil Nadu | 47 | 5 | 64 | 116 | 14 | 0 | 5 | 19 |
